# MPC1 Deficiency Promotes CRC Liver Metastasis via Facilitating Nuclear Translocation of *β*-Catenin

**DOI:** 10.1155/2020/8340329

**Published:** 2020-08-19

**Authors:** Guang-Ang Tian, Chun-Jie Xu, Kai-Xia Zhou, Zhi-Gang Zhang, Jian-Ren Gu, Xue-Li Zhang, Ya-Hui Wang

**Affiliations:** ^1^Shanghai Medical College of Fudan University, Shanghai 200032, China; ^2^State Key Laboratory of Oncogenes and Related Genes, Shanghai Cancer Institute, Renji Hospital, School of Medicine, Shanghai Jiao Tong University, Shanghai 200240, China; ^3^Department of Gastrointestinal Surgery, Renji Hospital, School of Medicine, Shanghai Jiao Tong University, Shanghai 200127, China

## Abstract

Accumulating evidence has pointed out that metastasis is the leading cause of death in several malignant tumor, including CRC. During CRC, metastatic capacity is closely correlated with reprogrammed energy metabolism. Mitochondrial Pyruvate Carrier 1 (MPC1), as the carrier of transporting pyruvate into mitochondria, linked the glycolysis and TCA cycle, which would affect the energy production. However, the specific role of MPC1 on tumor metastasis in CRC remains unexplored. Here, by data mining of genes involved in pyruvate metabolism using the TCGA dataset, we found that MPC1 was significantly downregulated in CRC compared to nontumor tissues. Similar MPC1 expression pattern was also found in multiple GEO datasets. IHC staining in both human sample and AOM/DSS induced mouse CRC model revealed significant downregulation of MPC1. What is more, we found that MPC1 expression was gradually decreased in normal tissue, primary CRC, and metastasis CRC. Additionally, poor prognosis emerged in the MPC1 low expression patients, especially in patients with metastasis. Following, functional tests showed that MPC1 overexpression inhibited the motility of CRC cells *in vitro* and MPC1 silencing enhanced liver metastases *in vivo*. Furthermore, we uncovered that decreased MPC1 activated the Wnt/*β*-catenin pathway by promoting nuclear translocation of *β*-catenin to mediate the expression of MMP7, E-cadherin, Snail1, and myc. Collectively, our data suggest that MPC1 has the potential to be served as a promising biomarker for diagnosis and a therapeutic target in CRC.

## 1. Introduction

Colorectal carcinoma (CRC), as one of the most common gastrointestinal malignancies, has developed the world's fourth most deadly cancer with a high rate of incidence and mortality [1, 2]. Liver metastasis, which is the most common form for CRC metastasis, is the leading cause of high mortality for this severe malignancy [2]. However, few clinical prevention and treatment measures could be available for tumor metastasis. Therefore, it is really urgent to develop new biomarkers and explore the underlying mechanism for CRC metastasis, and eventually develop new therapeutic strategies for CRC patients.

During cancer progression, metabolic reprograming with increasing glucose utilization is termed as Warburg affect, which is accompanied by altered pyruvate and mitochondrial metabolism [3]. The fate of pyruvate is the core manifestation to distinguish normal cells and tumor cells through metabolism. In normal cells, pyruvate was used for efficient ATP production directly into mitochondria. However, pyruvate was converted into lactate in cytosol despite of normoxic and hypoxic conditions in cancer cells [4]. This may be due to the impaired process of pyruvate from the cytosol into the mitochondrial matrix, which is a critical metabolic step linking glycolysis and mitochondrial oxidative phosphorylation. The Mitochondrial Pyruvate Carrier (MPC), a multimeric complex containing two distinct proteins MPC1 and MPC2, which is located in the inner mitochondrial membrane, is responsible for efficient mitochondrial pyruvate uptake [5]. Loss of MPC expression or activity blocks pyruvate entry into the TCA cycle, which results in a metabolism switch to increase glycolysis and the compensatory usage of glutamine [6, 7].

Existed studies have reported that MPC1 was related with immunoregulation, stemness, metabolism, cellular morphology, etc. [3, 8–10]. Currently, the important role of MPC1 was uncovered in several tumors. In CRC and esophageal squamous cell carcinoma, decreased MPC1 results in accelerated aerobic glycolysis and malignant progression [11, 12]. In lung adenocarcinoma, MPC1 deficiency accelerates lung adenocarcinoma progression through the STAT3 pathway [13]. In prostate cancer, MPC1 was reported to be involved in stemness and metabolism which regulated by COUPTFII [14, 15]. In renal cell carcinoma, hypoxia-induced loss of MPC1 enhanced the expression of MMP7 and MMP9 to promote cell invasion [16]. Collectively, these data suggested that MPC1 maybe serves as a suppressor to disrupt tumor malignancy. However, whether MPC1 is involved in CRC metastasis and the underlying mechanisms remain to be illustrated.

In the present study, we figured out the relationship between the MPC1 expression and CRC liver metastasis. We identified that decreased MPC1 was closely correlated with patient's metastasis, as well as led to poor prognosis. Functionally, MPC1 overexpression could attenuate the migration and invasion capacities of CRC cells both *in vitro* and *in vivo.* Mechanically, MPC1 suppressed CRC metastasis through mediating the Wnt/*β*-catenin signaling. Thus, our finding firstly revealed a critical role of MPC1 in CRC liver metastasis.

## 2. Materials and Methods

### 2.1. Data Mining

Seven GEO datasets (GSE21510, GSE5206, GSE20916, GSE9348, GSE89393, GSE67675, and GSE4183) and TCGA were used to analyze the MPC1 expression pattern in CRC. The primary data for TCGA datasets were downloaded https://www.cancer.gov/. The primary data for GEO datasets were downloaded at https://www.ncbi.nlm.nih.gov/geo. The OncoLnc database (http://www.oncolnc.org/) was used to detect the prognostic value of MPC1 in the TCGA cohort.

### 2.2. Patients and Clinical Specimens

In our study, a total of 392 patients containing paired CRC tissues and adjacent nontumor tissues were enrolled from the Department of Gastrointestinal Surgery, Renji Hospital, School of Medicine, Shanghai Jiao Tong University. Among them, 30 cases of liver metastases were collected. And only 225 cases were available with complete follow-up data for survival analysis. Informed consents were signed by all patients. The research was approved by the Research Ethics Committee of Renji Hospital and carried out in accordance with ethical standards as formulated in the Helsinki Declaration.

### 2.3. Cell Culture and Cell Transduction

Human CRC cell lines (HCT116, HT29, SW620, RKO, SW480, and Lovo) and mouse CRC cell line MC38 were gained from the Cell Bank of the Chinese Academy of Sciences (Shanghai, China). All cells were cultured in DMEM medium supplemented with 10% fetal bovine serum and 1% antibiotics at 37°C in a humidified incubator with 5% CO2.

MC38 cells were transfected with lentivirus containing a luciferase reporter plasmid. Stable transfection cells were screened with 5 *μ*g/ml blastisidin for 14 days, which termed MC38-Luc. In addition, one short hairpin RNA (shRNA) sequence against MPC1 was packaged as lentivirus and transfected into MC38-luc cells. Lovo and SW480 were transfected with lentivirus containing full-length human MPC1 cDNA or empty vehicle control. Stable transfection cells were screened under 2 *μ*g/ml puromycin for 14 days and verified by western blot. In those assays, all lentiviral transfections were performed in the presence of 6 *μ*g/ml polybrene.

### 2.4. Immunohistochemical (IHC)

The protocol of this assay and quantify the MPC1 protein expression level were performed according to previously reported [17]. Primary antibodies used as follows: MMP7 (YT2663, Immunoway), E-cadherin (14472, CST), Snail1 (ab53519, Abcam), and MYC (YP0861, Immunoway).

### 2.5. Cellular Immunofluorescence

Assays were performed according to the previous description [18]. Briefly, cells were incubated with antibodies against *β*-catenin (ab32572, Abcam) and incubated with Alexa 488-conjugated secondary antibody (1 : 200). The nuclei were stained with 4′,6-diamidino-2-phenylindole (DAPI).

### 2.6. Western Blotting

Whole-cell lysates or nuclear protein was extracted using a protein extraction buffer (Beyotime, Shanghai, China) or nucleoprotein extraction kit (Sangon Biotech, C500009), respectively. Proteins were resolved by SDS-PAGE and transferred onto nitrocellulose (NC) membranes using standard methods. Primary antibodies used as follows: MPC1 (ab74871, Abcam), *β*-catenin (ab32572, Abcam), lamin A/C (ab8984, Abcam), and GAPDH (ab9485, Abcam). Species-specific secondary antibodies used as follows: IRDye 680 Goat anti-Mouse IgG (LI-COR) and IRDye 800 Goat anti-rabbit IgG (LI-COR).

### 2.7. Real-Time PCR

Total RNA was extracted using Trizol (Takara) according to the manufacturer's instructions. cDNA was synthesized using the PrimeScript reverse transcription-polymerase chain reaction (RT-PCR) kit (Takara). The qPCR was performed using SYBR Green Master Mix (Roche). The 2 ^−*△*CT^ method was used to analyze the data and GAPDH was used as a loading control. The primer sequences are listed in Table 1.

### 2.8. Liver Metastasis Model

This study was performed in accordance with the recommendations in the Guide for the Care and Use of Laboratory Animals and relevant Chinese laws and regulations. The protocol was approved by the Institutional Animal Care and Use Committee (IACUC) of Shanghai Jiao Tong University. The MC38 cells transplanting luciferase-expressing were injected into the spleens of C57BL/6N mice (*n* = 5) with a concentration of 10^6^ cells/mouse. Two weeks later, the mice were killed and the liver metastasis tissues were harvested.

### 2.9. Luciferase Reporter Assay

The protocols of this assay were operated in accordance with previous reported [18]. 100 ng TOP reporter plasmid (Wnt/*β*-catenin signaling) or 100 ng FOP reporter plasmid (negative control of Wnt/*β*-catenin signaling) and 10 ng Renilla were mixed and transfected into CRC cells using Lipofectamine 2000. Dual-luciferase reporter assay system (Promega) was used to detect the firefly and Renilla luciferase activities.

## 3. Statistical Analyses

Data are shown as means ± SD. SPSS 20.0 (Chicago, IL, USA) and GraphPad Prism 5 software were used to manipulate statistical analyses. Kaplan-Meier method was used to calculate cumulative survival time. The chi-square test or Student's *t*-test was used for comparison between groups. *P* < 0.05 was considered as statistically significant.

## 4. Results

### 4.1. MPC1 Expression Was Aberrantly Decreased in CRC

**Figure 1:**
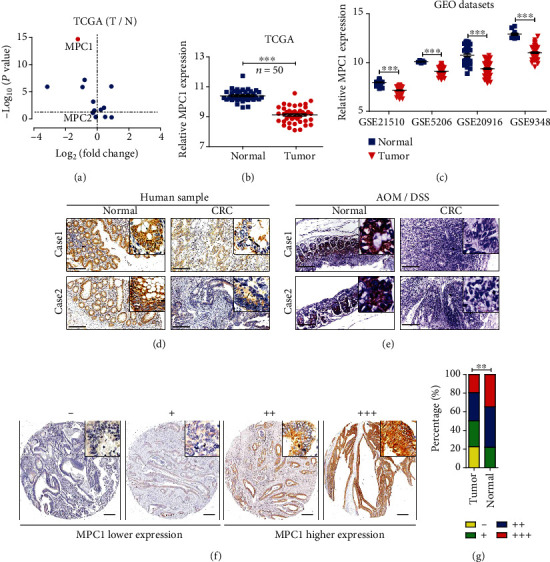
Expression pattern of MPC1 in CRC. (a) Volcano plot showed fold changes (*x*-axis) and corresponding *P* values (log_10_, *y*-axis) of pyruvate metabolism-related genes located in mitochondria analyzed in the TCGA dataset between paired normal and CRC samples (Student's *t*-test). (b) The comparison of MPC1 expression in tumor and matched normal tissues using the TCGA dataset (Student's *t*-test, ^∗∗∗^*P* < 0.001). (c) Expression analysis of MPC1 in tumors and corresponding normal tissue using four independent GEO datasets (GSE21510, GSE5206, GSE20916, and GSE9348) (Student's *t*-test, ^∗∗∗^*P* < 0.001). (d) IHC staining of MPC1 expression in matched CRC tumor and nontumor tissues (Scale bar: 200 *μ*m). (e) IHC staining of MPC1 expression in the AOM/DSS induced CRC mouse models and control animal (Scale bar: 200 *μ*m). (f) Representative IHC staining of MPC1 expression in tissue microarray from the Ren Ji cohort which contained CRC patients and paired adjacent normal tissue (*n* = 392), the tumor tissues were divided into two groups based on the expression level which scored as “-, +, ++, +++” (Scale bar: 500 *μ*m). (g) The percentage of tissue displaying different expression level of MPC1 in CRC tumor and adjacent nontumor tissues (Fisher's exact test, ^∗∗^*P* < 0.01).

Pyruvate is a pivotal intermediate in the process of cell metabolism, which connects glycolysis and the TCA cycle. To determine the potential maladjustment genes involved in pyruvate metabolism, which is located in mitochondria, as shown in Table 2, we analyzed the TCGA dataset containing 50 CRC and their normal counterparts. The results showed that multiple genes are significantly upregulated or downregulated in CRC (T) tissues compared to normal colon (N). Notably, MPC1 had a log2 (fold change) less than -1 (Figures 1(a) and 1(b)). As known to us, pyruvate translocation from the cytoplasm to the mitochondria is the first step into the TCA cycle, which needs MPC1/MPC2 heterodimer. In the analysis, no significant change was found in MPC2. Therefore, MPC1 was selected for further study. The expression pattern of MPC1 was further analyzed in five independent GEO datasets (GSE21510, GSE5206, GSE20916, GSE9348, and GSE4183). Consistently, we found that MPC1 was downregulated with statistical difference in CRC tissues (Figure 1(c)) and inflammatory tissue (Supplementary Figure 1) in comparison to their normal counterparts. Meanwhile, we found decreased MPC1 expression in human CRC tissues compared to their normal counterparts (Figure 1(d)). In addition, a similar phenomenon was revealed in AOM/DSS induced mouse CRC models (Figure 1(e)). Then, we evaluated the protein level of MPC1 used a tissue microarray containing 392 matching cancer and corresponding adjacent nontumor tissues which subjected IHC staining. The expression of MPC1 was scored as “-, +, ++, +++” based on the staining area and intensity (Figure 1(f)). We found that more lower MPC1 expression (score as “-” and “+”) was presented in CRC tissues (Figure 1(g)). Overall, these results revealed that MPC1 was disrupted during CRC.

### 4.2. Decreased MPC1 Enhanced Tumor Metastasis Capability and Predicated Poor Prognosis in CRC

**Figure 2:**
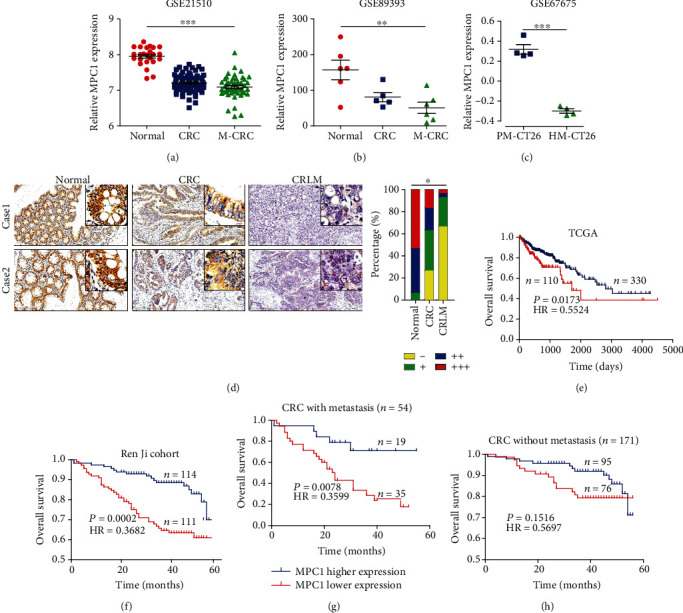
Decreased MPC1 enhances tumor metastasis and predicts poor prognosis in CRC. (a, b) Data mining showed MPC1 expression was gradually decreased in normal tissue, primary CRC, and liver lesion of metastatic CRC (M-CRC) from two independent GEO datasets (One-way ANOVA, A, GSE21510, ^∗∗∗^*P* < 0.001; B, GSE89393, ^∗∗^*P* < 0.01). (c) Data from GSE67675 revealed that MPC1 expression was lower in high liver metastasis CT26 cells (HM-CT26) than that in poor liver metastasis CT26 cells (PM-CT26) (Student's *t*-test, ^∗∗∗^*P* < 0.001). (d) Gradually decreased MPC1 expression was presented in normal tissue, primary CRC, and liver metastasis CRC (CRLM) tissue (Scale bar: 200 *μ*m) (*n* = 30, Fisher's exact test, ^∗^*P* < 0.05). (e) Kaplan-Meier overall survival (OS) curves in the TCGA dataset of CRC patients according to the mRNA expression of MPC1, the lower quartile value of expression was utilized as a cut-off (log-rank test, *P* = 0.0173). (f) Kaplan-Meier OS curve for the MPC1 expression in the Ren Ji cohort (log-rank test, *P* = 0.0033). (g) Kaplan-Meier OS curve for the MPC1 expression in patients with metastasis (log-rank test, *P* = 0.0078). (h) Kaplan-Meier OS curve for the MPC1 expression in patients without metastasis (log-rank test, *P* = 0.1516).

It is well known that CRC is one of the most malignant tumors given its strong metastasis ability, so we tried to figure out whether the MPC1 expression was correlated with metastasis. We found that lower MPC1 expression was closely correlated with metastasis (*P* = 0.009), lymph node invasion (*P* = 0.003), and TNM stage (*P* = 0.001), which revealed by the analysis between the clinical significance and MPC1 expression in CRC (Table 3). Next, to illuminate the expression pattern of MPC1 in different process of CRC, data mining was carried out by two independent GEO datasets (GSE21510 and GSE89393), which contained normal tissue, primary CRC, and metastatic lesion in the liver (M-CRC). As shown in Figures 2(a) and 2(b), MPC1 expression was gradually downregulated in patients with an increase in metastasis ability. A similar result was found in mice cells CT26 with high liver metastasis (HM-CT26) or poor liver metastasis (PM-CT26) (Figure 2(c)), which isolated by in vivo selection in an orthotopic mouse model of colon cancer metastasis to the liver. Further analysis showed that MPC1 protein expression was gradually downregulated in normal tissue, primary CRC, and liver metastasis CRC (CRLM) tissues (Figure 2(d)). Furthermore, survival analysis showed that patients with lower MPC1 expression had a worse outcome compared to the patients with higher MPC1 expression using the TCGA cohort (Figure 2(e)) and Ren Ji cohort (Figure 2(f)). Additionally, among patients with metastasis, worse prognosis was emerged in the MPC1 low cases (Figure 2(g)), while no significant association was observed in patients without metastasis (Figure 2(h)).

### 4.3. MPC1 Overexpression Impaired CRC Cells Motility Both *In Vitro* and *In Vivo*

**Figure 3:**
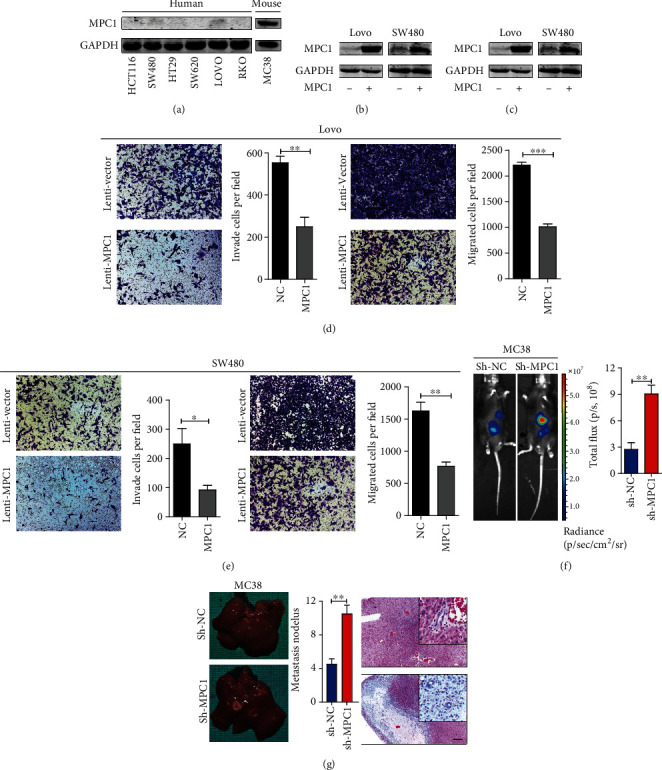
MPC1 overexpression inhibits the motility of CRC cells *in vitro and in vivo*. (a) MPC1 expression in human and mouse CRC cell lines examined by western blot. GAPDH serves as loading control. (b) MPC1 overexpression in Lovo and SW480 cells. (c) MPC1 silencing by sh-RNA-MPC1 in mouse MC38 cells. (d, e) Transwell assays showed that upregulated MPC1 suppressed the invasion and migration ability of Lovo (d) and SW480 (e) cells. Quantification of invaded and migrated cells was performed for five randomly selected fields, values are means ± SD. (f) Representative bioluminescence photograph of mice spleen implanted with luciferase-expressing MC38 cells treated with sh-MPC1 or control vector, total flux was quantified by the IVIS system to verify the ability of liver metastasis. (g) Representative image of liver metastases and quantified by the nodules in mice inoculated with MC38 cells treated with sh-MPC1 or control vector, as well as representative images of H&E staining of the liver metastatic lesions. Scale bar: 100 *μ*m. Student's *t*-test, ^∗^*P* < 0.05, ^∗∗^*P* < 0.01, ^∗∗∗^*P* < 0.001.

To evaluate the role of MPC1 on the motility of CRC cells, the transwell assay was performed. Firstly, we examined low MPC1 protein expression in human and high MPC1 protein expression in mouse MC38 CRC cells by western blot (Figure 3(a)). MPC1-overexpressing stable cell lines were established using a lentivirus carrying the MPC1 gene in Lovo and SW480 cells. And the overexpression efficiency was confirmed by immunoblots (Figure 3(b)). MPC1 knockdown in Luc-MC38 cells were established and verified by WB (Figure 3(c)). MPC1 overexpression exhibited significantly weaker migration and invasion ability than the control cells in both Lovo (Figure 3(d)) and SW480 (Figure 3(e)) cells. Following, the liver metastasis model of CRC was established by spleen orthotopically injecting MC38 cells transplanting luciferase-expressing, which would simulate MC38 metastasis to the liver through splenic vein-portal vein. The results revealed that the MPC1 knockdown promoted MC38 cells metastasis to the liver through detecting the luminescence intensity monitored by bioluminescence imaging (Figure 3(f)). Notably, the number of metastatic liver nodules in the MPC1 silencing group was smaller than that in the control group (Figure 3(g)). Histological examination also proved that MPC1 knockdown decreased the metastatic potential of CRC *in vivo* (Figure 3(g)).

### 4.4. Decreased MPC1 Activated the Wnt/*β*-Catenin Pathway by Promoting Nuclear Translocation of *β*-Catenin

**Figure 4:**
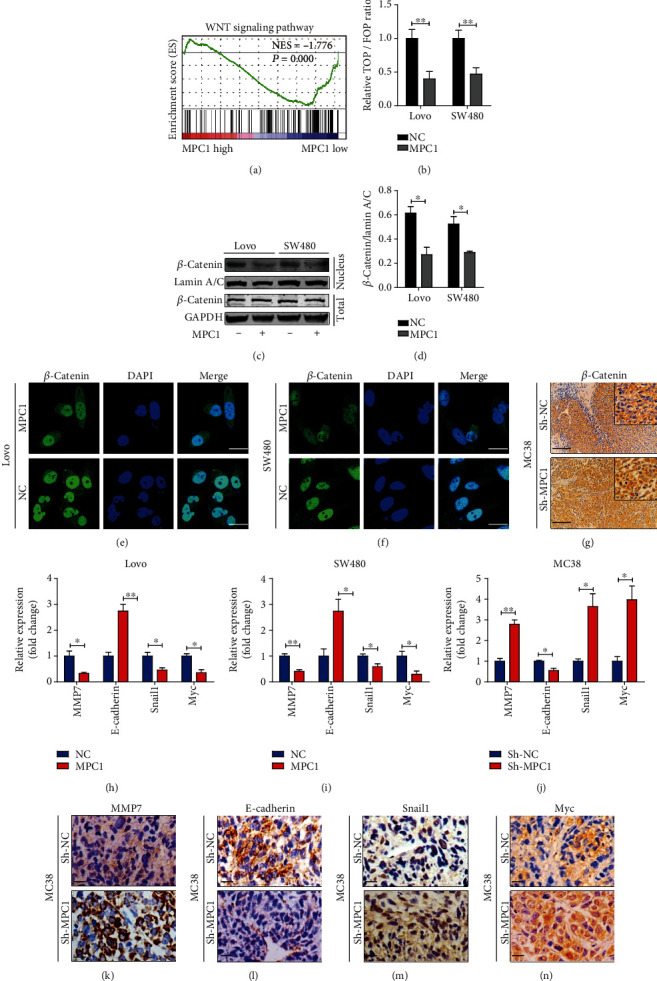
Decreased MPC1 activates the WNT/*β*-catenin pathway by promoting nuclear translocation of *β*-catenin. (a) GSEA analysis of MPC1 expression in CRC using the TCGA dataset. NES: Normalized Enrichment Score. (b) Luciferase reporter gene assay of CRC cells treated with MPC1 overexpression or not. (c) The expression of total *β*-catenin and nuclear *β*-catenin was detected in control and MPC1-overexpression CRC cells, respectively. GAPDH and lamin A/C were used as the loading control of total and nuclear protein, respectively. (d) The gray value analysis of nuclear *β*-catenin in MPC1-overexpression cells and control cells. (e, f) MPC1 overexpression could inhibit the nuclear translocation of *β*-catenin in CRC cells. Scale bar: 100 *μ*m. (g) IHC staining of *β*-catenin in mouse liver metastatic lesions inoculated with MC38 cells treatment with sh-MPC1 or control vector. Scale bar: 200 *μ*m. (h, i) Relative mRNA expression level of *β*-catenin target genes in CRC cells with MPC1 overexpression or control vector. (j) Relative mRNA expression level of *β*-catenin target genes in MC38 cells with sh-MPC1 or control vector. (k–n) Relative protein expression level of *β*-catenin target genes in mouse liver tissue detected by IHC. Scale bar: 50 *μ*m. Student's *t*-test, ^∗^*P* < 0.05, ^∗∗^*P* < 0.01.

To further explore the underlying mechanism of MPC1-mediated inhibition of CRC metastasis, the TCGA database was used to perform GSEA analysis. The results indicated that MPC1 was involved in the Wnt/*β*-catenin signaling when set the mRNA expression median as a cutoff (Figure 4(a)). And dual-luciferase reporter gene assay revealed that MPC1 overexpression obviously inhibited the activity of Wnt/*β*-catenin pathway (Figure 4(b)), which confirmed the result above. We then tested the distribution changes of *β*-catenin in nuclear and cytoplasmic, which was a crucial step in Wnt/*β*-catenin pathway. As shown in Figure 4(c), no significant difference was emerged in the total amount of *β*-catenin between MPC1 overexpression cells and the control cells (Figure 4(c)). However, obviously decreased distribution of nuclear *β*-catenin was presented in MPC1 overexpression cells compared to that in the control cells, as revealed by the stronger gray corresponding to *β*-catenin (Figures 4(c) and 4(d)). Consistently, immunofluorescence (IF) staining showed that MPC1 overexpression weakened nuclear *β*-catenin localization in both Lovo and SW480 cells when compared to control cells (Figures 4(e) and 4(f)). A Similar phenomenon was observed in mouse liver metastasis tissues by IHC (Figure 4(g)). Following, qPCR analyses displayed some downstream target genes of *β*-catenin, such as MMP7, E-cadherin, Snail1, and MYC. Obviously increased in E-cadherin and decreased in MMP7, Snail1, and MYC were observed after the MPC1 overexpression in both Lovo and SW480 cells compared to that in the control cells (Figures 4(h) and 4(i)). Meanwhile, the opposite trend was observed in MC38 cell after MPC1 knockdown (Figure 4(j)), and similar results were observed in mouse liver tissue detected by IHC (Figures 4(k)–4(n)). Taken together, the data above indicated that MPC1 mediated CRC cell metastasis through the Wnt/*β*-catenin pathway.

## 5. Discussion

Accumulating evidences have shown that reprogrammed energy metabolism conduces to the tumor malignant properties, including enhanced CRC liver metastatic capacity [19–21]. Mitochondria, as the primary site of energy production, regulate the pyruvate metabolism under both physiologic and pathologic conditions. The first step of the TCA cycle is mediated by MPC, which transports pyruvate into mitochondrial from cytoplasm [3]. In the beginning, TCGA was used to analyze the relative genes involved in pyruvate metabolism, which is located in mitochondria. The results revealed that MPC1 expression was significantly downregulated in CRC tissues. Meanwhile, the GEO datasets analysis, as well as, IHC staining on CRC patients' tissue and mouse models confirmed this trend. These phenomena may indicate that loss of MPC activity enhanced tumorigenic glucose utilization by blocking mitochondrial pyruvate uptake and oxidation. Interestingly, in the course of data analysis, we found that the expression of MPC1 was decreased at the stage of intestinal inflammation, which was not different from that in tumor tissue. MPC1 has been reported to be involved in immune regulation of peripheral T cell homeostasis through metabolic regulation [8]. And a decrease in MPC1 was found at the earliest stages of CRC [22]. Hence, we suspect that MPC1 is involved in bowel inflammation to tumorigenesis. And more studies need to be devised to illustrate this process.

Following, in the analysis between the clinical significance and MPC1 expression in CRC, we found that MPC1 expression was especially correlated with metastasis. Inspired by this, we detected the MPC1 expression pattern in normal tissue, primary CRC, and metastasis CRC by GEO datasets and patients' tissue. All results revealed that gradually downregulated MPC1 was in patients with an increase in metastasis ability. Survival analysis indicated that worse outcome was presented in patients with lower MPC1 expression, especially in patients with metastasis. Additionally, function assays verified that MPC1 overexpression could attenuate the migration and invasion capacities of CRC cells *in vitro* and MPC1 knockdown could enhance the metastasis capacity *in vivo.* And existing studies have revealed that the MPC1 was participated in metastatic dissemination of PGC1*α*-transduced cholangiocarcinoma through elevating reactive oxygen species (ROS) production [10]. Besides, MPC inhibitor UK5099 treatment could trigger strong invasive capacity through blocking pyruvate translocation into the mitochondria so as to attenuate mitochondrial oxidative phosphorylation and trigger aerobic glycolysis [12]. These phenomena together with our results indicate that MPC1 could act as a tumor suppressor through inhibiting tumor metastasis. And existing studies have shown that MPC1 could alter the maintenance and fate of stem cells through regulating cancer metabolism in CRC [11]. However, the relationship between the metabolism and tumor metastasis regulated by MPC1 was not being mentioned. Thus, further evidence should be received to confirm this.

Subsequently, the underlying mechanism of MPC1 in regulating metastasis was explored. For this purpose, GSEA analysis was performed. The results indicated that MPC1 was involved in the Wnt/*β*-catenin signaling. The next series of experiments also confirmed that MPC1 could mediate the WNT/*β*-catenin pathway by redistribution of *β*-catenin. Previous reports showed that cytoplasmic *β*-catenin phosphorylated in N-terminally localized to sites of cell-cell contact is associated with E-cadherin and was required for intact cell-cell adhesions, without any change detected in the levels of total *β*-catenin [23–25]. Simultaneously, cell–cell adhesion based on cadherin binding with *β*-catenin limited Wnt signals [24]. In addition, *β*-catenin was reported to interact with USP9x to inhibit the degradation of *β*-catenin through the deubiquitination of *β*-catenin in breast cancer [26]. A constitutive IRS1 and *β*-catenin protein interaction activated MYC expression in Acute Lymphoblastic Leukemia Cells [27]. In HCC, *β*-catenin was reported to interact with Yap1 to lead to rapid tumorigenesis [28]. Hence, it is reasonable to guess that accumulated cytoplasmic *β*-catenin maybe crosstalk with other genes or involved in other biological processes. What is more, some downstream target genes of *β*-catenin, such as MMP7, E-cadherin, Snail1, and myc, were changed in expression. As known to us, MMP-7 is a member of the proteolytic enzyme family, which promotes the invasion and metastasis of tumor cells by degrading the basement membrane and extracellular matrix [29]. And previous studies had evidenced for involvement of MMP-7 activation in colorectal cancer liver metastases [30, 31]. E-cadherin and Snail1 were considered as the epithelial-mesenchymal transition (EMT) marker, which was involved in metastasis of malignant tumor [32]. Moreover, E-cadherin was reported to be involved in cell-cell junction to regulate cancer invasion and metastasis [33, 34]. And the GSEA analysis also revealed that MPC1 could affect the cell-cell contacts (data not shown). As described previously, MMP-7 could facilitate morphological transition by cleaving E-cadherin [35]. The communication between the cells is disrupted when E-cadherin was shredded, leading to destructed cell adhesion and induction of EMT, followed by increased cell migration [36]. Inspired by this, we assumed that MPC1 could mediate tumor cell motility through affecting MMP7 activity, cell-cell contacts, and EMT. However, further studies need to be performed to clarify the detailed underlying mechanisms.

## 6. Conclusions

In conclusion, we firstly demonstrated that decreased MPC1 was closely correlated with patient's metastasis, as well as led to poor outcome. Moreover, MPC1-driven nuclear translocation of *β*-catenin contributed to CRC cell motility. This means that MPC1 has the potential to be a diagnostic biomarker and therapeutic target for metastasis patients.

## Figures and Tables

**Table 1:** Sequences of primers used for real-time PCR.

Primer	Sequence 5′-3′
MMP7 forward	GAGTGAGCTACAGTGGGAACA
MMP7 reverse	CTATGACGCGGGAGTTTAACAT
E-cadherin forward	ATGAGTGTCCCCCGGTATCTTC
E-cadherin reverse	ACGAGCAGAGAATCATAAGGCG
Snail1 forward	GTATCCAGAGCTGTTTGGA
Snail1 reverse	AACATTTTCCTCCCAGGCC
Myc forward	ATCACAGCCCTCACTCAC
Myc reverse	ACAGATTCCACAAGGTGC
GAPDH forward	CTGGGCTACACTGAGCACC
GAPDH reverse	AAGTGGTCGTTGAGGGCAATG

**Table 2:** Related enzyme and carrier of pyruvate analyzed in this study.

Gene	Description	Location
PDHA1	Pyruvate dehydrogenase (lipoamide) alpha 1	Mitochondrion matrix.
PDHB	Pyruvate dehydrogenase (lipoamide) beta	Mitochondrion matrix.
PDK4	Pyruvate dehydrogenase kinase, isozyme 4	Mitochondrion matrix.
PDHX	Pyruvate dehydrogenase complex, component X	Mitochondrion matrix.
MPC2	Mitochondrial pyruvate carrier 2	Mitochondrion inner membrane
PDK2	Pyruvate dehydrogenase kinase, isozyme 2	Mitochondrion matrix.
PDK1	Pyruvate dehydrogenase kinase, isozyme 1	Mitochondrion matrix.
PDK3	Pyruvate dehydrogenase kinase, isozyme 3	Mitochondrion matrix.
MPC1	Mitochondrial pyruvate carrier 1	Mitochondrion inner membrane
PC	Pyruvate carboxylase	Mitochondrion matrix.
PDP1	Pyruvate dehydrogenase phosphatase catalytic subunit 1	Mitochondrion matrix.
PDPR	Pyruvate dehydrogenase phosphatase regulatory subunit	Mitochondrion matrix.
PDHA2	Pyruvate dehydrogenase (lipoamide) alpha 2	Mitochondrion matrix.
PDP2	Pyruvate dehydrogenase phosphatase catalytic subunit 2	Mitochondrion matrix.
PKLR	Pyruvate kinase, liver and RBC	Mitochondrion

**Table 3:** Clinicopathological correlation of MPC1 expression in the Ren Ji CRC cohort.

Clinicopathological feature	Expression of MPC1		
	Low	High	*P* value (*χ*^2^ test)
Age (years)			
<60	95	96	0.766
≥60	103	98	
Gender			
Male	112	120	0.287
Female	86	74	
Metastasis			
Yes	52	30	0.009
No	146	164	
Lymph node invasion			
Yes	106	75	0.003
No	91	119	
Tumor size			
<5 cm	99	108	0.388
≥5 cm	94	86	
TNM stage			
I	15	20	0.001
II	58	92	
III	74	52	
IV	51	30	
KRAS mutation			
Mutation	33	40	0.246
No mutation	51	43	

## Data Availability

The data used to support the findings of this study are available from the corresponding author upon request.

## Abbreviations

CRC: Colorectal carcinoma
MPC: Mitochondrial pyruvate carrier
EMT: Epithelial-mesenchymal transition
GSEA: Gene set enrichment analysis.

## Supplementary Materials

Supplementary Materials “Supplementary Figure 1: expression analysis of MPC1 in normal, IBD, and tumor tissues using the GSE4183 dataset. One-way ANOVA was used to analyze the statistical differences between IBD, adenoma, and CRC tissues. ns: No significance. Student's *t*-test, ^∗∗∗^*P* < 0.001, ^∗∗^*P* < 0.01, ^∗^*P* < 0.05).”
