# Supplementary material for: MPC1 Deficiency Promotes CRC Liver Metastasis via Facilitating Nuclear Translocation of β-Catenin
Source: J Immunol Res. 2020 Aug 19;2020:8340329. doi: 10.1155/2020/8340329 (PMC7439788; doi:10.1155/2020/8340329)
Supplement: Supplementary Materials — “Supplementary Figure 1: expression analysis of MPC1 in normal, IBD, and tumor tissues using the GSE4183 dataset. One-way ANOVA was used to analyze the statistical differences between IBD, adenoma, and CRC tissues. ns: No significance. Student's t-test, ∗∗∗P < 0.001, ∗∗P < 0.01, ∗P < 0.05).” [file 8340329.f1.docx]

**MPC1 deficiency promotes CRC liver metastasis via facilitating nuclear translocation of β-catenin**

Guang-Ang Tian ^a,b,1^, Chun-Jie Xu ^c,1^, Zhi-Gang Zhang ^b^, Jian-Ren Gu ^a,b^, Xue-Li Zhang ^b,^**, Ya-Hui Wang ^b,^*

^a^ Shanghai Medical College of Fudan University, Shanghai 200032, PR China

^b^ State Key Laboratory of Oncogenes and Related Genes, Shanghai Cancer Institute, Renji Hospital, School of Medicine, Shanghai Jiao Tong University, Shanghai 200240, PR China

^c^ Department of Gastrointestinal Surgery, Renji Hospital, School of Medicine, Shanghai Jiao Tong University, Shanghai 200127, PR China

* / ** Corresponding author. State Key Laboratory of Oncogenes and Related Genes,

Shanghai Cancer Institute, Ren Ji Hospital, School of Medicine, Shanghai Jiao Tong

University, 800 Dongchuan Road, Shanghai 200240, PR China

E-mail addresses: [yhwang@shsci.org](mailto:yhwang@shsci.org) (Ya-Hui Wang) [xlzhang@shsci.org](mailto:xlzhang@shsci.org) (Xue-Li Zhang)

^1^ These authors contributed equally to this work.

Supplementary Fig.1


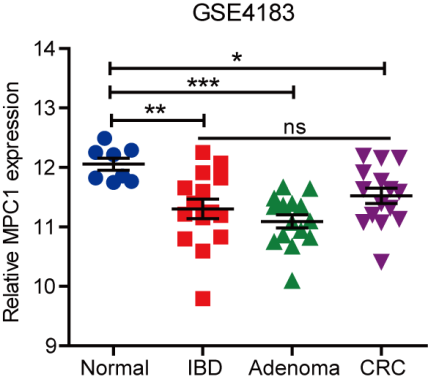


Supplementary Fig.1 Expression analysis of MPC1 in normal, IBD and tumor tissues using GSE4183 dataset. One-way ANOVA was used to analyze the statistical differences between IBD, adenoma and CRC tissues. ns: no significance. student's t-test, ***P < 0.001, **P < 0.01, *P < 0.05).
